# Influenza Vaccination in Children Younger than 5 Years in the Region of Murcia (Spain), a Comparative Analysis among Vaccinating and Non-Vaccinating Parents: Data from the FLUTETRA Study

**DOI:** 10.3390/vaccines12020192

**Published:** 2024-02-13

**Authors:** Jaime Jesús Pérez Martín, Matilde Zornoza Moreno, Francisca Isabel Tornel Miñarro, María Cruz Gómez Moreno, María del Carmen Valcárcel Gómez, Marta Pérez Martínez

**Affiliations:** Affiliation Vaccination Program, Prevention and Health Protection Service, Public Health and Addictions Directorate General, Region of Murcia Ministry of Health, 30008 Murcia, Spain; jaimej.perez@carm.es (J.J.P.M.); franciscai.tornel@carm.es (F.I.T.M.); mariac.gomez16@carm.es (M.C.G.M.); marivalgo@hotmail.com (M.d.C.V.G.); martuu1998@gmail.com (M.P.M.)

**Keywords:** influenza, vaccination, intranasal, satisfaction and acceptability, Spain

## Abstract

The high burden of influenza in children has driven numerous countries towards universal vaccination of healthy children from 6 to 59 months of age. The Region of Murcia was one of the pioneer Spanish regions to conduct a universal vaccination campaign and to use live-attenuated intranasal vaccine (LAIV) if age appropriate. This study aims to evaluate the parents’ likeliness to vaccinate their children and to compare the profile of vaccinating/non-vaccinating parents. This study was designed as a prospective, real-world, survey-based data collection in the 2022–2023 season campaign. This study’s sample was selected from those children whose information was available in the local Public Health System databases PERSAN and VACUSAN. Children received LAIV or intramuscular vaccine (IIV) depending on their age as per standard practice. The parent self-vaccination/intention to vaccinate themselves in this campaign (OR = 4.75), the compliance with the official vaccination schedule (OR = 3.41), and the prescription of antibiotics more than twice in the previous year (OR = 2.24) were strongly associated with children’s vaccination. Overall, vaccinating parents were very satisfied with the vaccine (IIV: 67.5% vs. LAIV: 68.8%, *p* = 0.320), and most parents would rather have their children vaccinated with LAIV for the next campaign (43.0%). The main reasons for vaccinating were to protect the child (LAIV: 85.9% vs. IIV: 89.4%), and the predominant reasons for not vaccinating were a lack of healthcare professional recommendation (30.9%), and lack of information about the vaccination campaign (21.5%) and the vaccine itself (21.0%). The clinical context of parents and children was determinant in decision making, which was also influenced by the presence or absence of recommendation by healthcare professionals. Parents were generally very satisfied with the vaccine and showed their preference towards LAIV for future campaigns.

## 1. Introduction

Seasonal influenza is still one of the most important concerns of health systems due to its clinical and economic impact in the general population, focused specifically on adult risk groups, and children [1,2,3]. Children under 5 years of age manifest a high disease burden and represent an important primary vector of influenza transmission [4,5,6]. In Spain, the cumulative rate of hospitalization due to severe confirmed influenza (defined as clinically confirmed influenza syndrome requiring hospitalization due to the severity of the clinical features) observed in the last few seasons places children younger than 5 years, along with older adults (≥65), as the most hospitalized patients. The most recent data available (season 2019–2020) show a hospitalization rate of 52.4 per 100,000 inhabitants in children younger than 5 years old. Moreover, the average number of children from 0–4 years hospitalized over the course of seasons rose to 4239, more than double the number of patients aged 5–14. Among children 0 to 5 years old hospitalized with confirmed severe cases of influenza, 68.4% of cases hospitalized and 56.1% admitted into the Intensive Care Unit (ICU) did not present risk factors [7]. Moreover, a retrospective study evaluating data from hospital admissions in children younger than one year of age in Spain from 2009 to 2017 reported that 54.5% of hospitalizations were due to influenza, with an average hospitalization rate of 160.17/100,000 inhabitants (excluding the 2009–2010 H1N1 pandemic year) [8].

Prior to 2022, the pediatric influenza vaccination recommendations in Spain were limited to children older than 6 months with risks of complications or living with relatives at risk, along with the traditionally defined risk groups (healthcare professionals, subjects older than 65 years, pregnant women, patients suffering from respiratory diseases, etc.) [7]. Despite these recommendations, the pediatric vaccination rate in groups at risk was still a target, being significantly lower than that of adult vaccination, and with remarkable differences among autonomous communities [9,10,11]. Low vaccination coverage was observed not only in Spain, but also in other European regions [12,13,14,15]. This may be attributed to many causes: unawareness of the disease burden in children, concerns about the vaccine efficacy and/or safety, or lack of knowledge about the seasonal vaccination program, as reported in studies interviewing parents [13,16,17].

According to the World Health Organization (WHO) and the European Center for Disease Prevention and Control (ECDC), the Spanish National Immunization Technical Advisory Group recommended the systematic vaccination of healthy children from 6 to 59 months of age in 2022, and it was included in the official vaccination schedule in 2023 [7,18]. This recommendation has been also supported by the Vaccine Advisory Committee of the Spanish Association of Pediatrics since 2022–2023. The Region of Murcia, along with other Spanish regions—Andalusia and Galicia—were pioneers of systematic vaccination in the 2022–2023 campaign [19,20].

The non-invasive Live-Attenuated Influenza Vaccines (LAIVs) has changed the paradigm of vaccination since their approval, and these has been gradually introduced into the vaccination campaigns promulgated by national health systems, more specifically, the quadrivalent LAIV Fluenz^®^ Tetra, which is currently the only approved LAIV in Europe [21,22]. Many studies have evaluated the acceptance of LAIVs among parents and healthcare professionals, and compared it with that of traditional injectable vaccines, obtaining favorable results toward LAIV. The RELATIVES study, a pilot open-label trial conducted in schools of Peterborough County-City Public Health Unit (Canada) in the 2013–2014 season, estimated that parents felt more comfortable with their children receiving a LAIV, and that a higher percent of them preferred LAIV for the next campaign [16]. More recently, two observational studies conducted in Italy (Bologna and Milan, respectively) evaluated parents’ intention of vaccination and revaccination. Data from the Bologna cohort stated that most parents open to vaccination would prefer a non-injectable vaccine for future seasons, and many of the hesitant parents would reconsider vaccination if the vaccine were administered without a needle injection [13]. Most parents in the Milan cohort reported that they were very satisfied with the LAIV, and they would agree to revaccinate their children again in the next campaign [23].

In Spain, the Region of Murcia was one of the pioneer autonomous communities vaccinating children from 6 to 59 months in the 2022–2023 campaign, and the only one introducing LAIV as the election vaccine for children aged 24–59 months, except where contraindicated. For this pilot experience, the desired vaccination coverage was 50% of the target population [20].

Due to the novelty of universal influenza vaccination for children in Spain, and specifically in the Region of Murcia as one of the pioneer Autonomous Communities, there is no register of vaccination coverage after the last recommendations. Accordingly, we have designed the FLUTETRA study to obtain the first results of pediatric vaccination status in the 2022–2023 season. Furthermore, we intend to present a detailed and updated comparison of the profile of vaccinating and non-vaccinating parents and to explore the factors influencing the parents’ decision-making process. Finally, we aim to know the opinion of vaccinating parents on the vaccines received by their children. It is expected that the data discussed in this work will play an important role in the addition of other autonomous communities to the universal vaccination plan, thus contributing toward the national vaccination coverage, and even to other European regions.

## 2. Materials and Methods

### 2.1. Study Design and Participants

FLUTETRA is a prospective real-world data (RWD) study sponsored by the Service of Prevention and Health Protection of the Region of Murcia’s Ministry of Health (Spain), as part of the Spanish National Health System (Sistema Nacional de Salud, SNS). This study was conducted according to the guidelines of the Declaration of Helsinki and approved by the Ethics Committee of Hospital Clínico Universitario Virgen de la Arrixaca. In accordance with local regulations, the Ethics Committee approved an exemption from written informed consent.

To perform this analysis, parents of pediatric patients from 6 to 59 months of age during the 2022–2023 seasonal vaccination campaign in the Region of Murcia were voluntarily enrolled as study subjects. Data were consecutively collected from parents/legal representatives of children registered in PERSAN, the data management program of the population database in Murcia. Children with an influenza vaccine dose registered in VACUSAN (data management program of the Region of Murcia vaccination registry) from 2 November 2022 to 10 February 2023 were consecutively enrolled. In addition, parents of non-vaccinated children from 6 to 59 months of age were enrolled at the end of the campaign.

### 2.2. Treatment and Procedures

The vaccination process followed the local standard of care, primarily administering two different vaccines according to the subject’s age; children from 24 to 59 months received Fluenz^®^ Tetra LAIV (AstraZeneca UK Limited), and children aged 6 to 23 months received Influvac^®^ Tetra inactivated intramuscular vaccine (IIV) (Mylan IRE Healthcare Limited). Those children from 24 to 59 months of age with specific contraindications to LAIV received IIV Flucelvax Tetra^®^ (Seqirus Netherlands B.V.) [20].

Vaccinating parents/legal representatives accessed the study survey through a link sent by SMS 7 days after vaccination, and non-vaccinating parents/legal representatives received the survey in the same way at the end of the vaccination campaign.

### 2.3. Assessments and Endpoints

This study’s primary endpoint was to evaluate the parents’ likeliness to vaccinate their children. The secondary objectives analyzed in this work were to describe the sociodemographic characteristics of parents/legal representatives of vaccinees vs. non-vaccinees, and to evaluate parents’/legal representatives’ attitude towards vaccination.

### 2.4. Statistical Methods

A descriptive analysis was performed to describe all demographic and relevant medical history of children and parents. Descriptive analyses were performed by measures of central tendency and dispersion and counts and percentages to report qualitative variables, along with a 95% confidence interval (CI).

Comparative analyses were conducted between vaccinated and non-vaccinated children, and between those receiving IIV vs. LAIV. Fisher’s exact test was performed in order to compare relative frequencies, whereas the non-parametric Mann–Whitney U test was performed to compare ordinally scaled variables (i.e., age groups) between two groups.

A multivariable logistic regression analysis of potential factors independently associated with vaccination decision making was conducted in a binary model that plots the probability of being vaccinated against the variables of interest. For this purpose, the following covariates were assessed: prematurity, number of siblings, children’s vaccination against influenza in the last campaign, presence of acute infection disease during the previous year, frequency of antibiotic prescription, kindergarten attendance, up-to-date vaccination schedule, administration of non-funded vaccines, cohabitants with chronic disease and/or older than 60 years, personal opinion on childhood diseases causing most hospitalizations, parent’s vaccination against influenza in both current and past campaigns, and parent’s chronic disease. Variables with *p* < 0.2 in the univariable logistic regression analysis were considered significant and included in a multivariable model with stepwise selection method, and odds ratio (OR) and 95% CIs were calculated. Test results with *p* < 0.05 were considered statistically significant. The ORs provided in this analysis are graphically presented in figures to ease the interpretation and comparation of the results.

IBM SPSS Statistics version v9.4 (SAS Enterprise Guide v8.3) was used for all the analyses.

## 3. Results

### 3.1. Descriptive and Comparative Analysis of the Study Population

At the end of the vaccination campaign, data from 9999 parents/legal representatives was collected; 4971 subjects vaccinated their children and 5028 did not (see Supplementary Figure S1 for further details). Demographic and clinical data from both vaccinated children (VC) and non-vaccinated children (NVC) are displayed in Table 1. Overall, the data revealed a majority of Spanish children (98.8%) with a slight predominance of the male sex (51.8%). VC showed significatively higher rates of prematurity (8.7% vs. 6.4%, *p* < 0.001), last-year acute infections requiring medical attention (54.3% vs. 46.5%, *p* < 0.001), and frequency of last-year antibiotic prescriptions (none: 38.7% vs. 47.4%; once: 31.0% vs. 30.9%; twice: 17.2% vs. 13.9%, more than twice: 13.2% vs. 7.8%; *p* < 0.001). Moreover, children vaccinated in this campaign showed higher rates of compliance with the vaccination schedule (99.7% vs. 98.7%, *p* < 0.001), non-funded vaccines (88.3% vs. 80.1%, *p* < 0.001), and vaccination against influenza in the last campaign (12.6% vs. 4.6%, *p* < 0.001). Regarding the children’s context, NVC had more siblings (2 siblings: 13.6%, <2 siblings 4.0%), and presented remarkably higher kindergarten attendance (VC: 58.9% vs. NVC: 70.3%; *p* < 0.001).

Clinical and demographic data from parents/legal representatives who answered the survey are displayed in Table 1. The comparative analysis showed statistically significant differences in the parent’s sociodemographic profiles regarding age, sex, country of origin, and education. Most survey responders were Spanish (vaccinating parents [VP], 91.0% vs. non-vaccinating parents [NVP] 86.5%; *p* < 0.001), women (92.3% vs. 83.1%; *p* < 0.001) and aged 30–39 years old (61.5% vs. 55.3%; *p* < 0.001). Overall, the parents had a heterogeneous educational background, with most of them having completed higher education in both groups (VP, 57.4% vs. NVP, 55.9%, *p* < 0.001). VP had a significatively higher presence of chronic disease (18.5% vs. 15.5%; *p* < 0.001) and a higher percentage of them cohabitate with old and/or chronically ill relatives (15.0% vs. 9.6%; *p* < 0.001). Moreover, there were more VP who received vaccination against influenza in both the present (53.2% vs. 18.4%, *p* < 0.001) and the past campaigns (42.6% vs. 24.6%, *p* < 0.001) in comparison to NVP.

Among VC, 1437 received IIV and 3531 LAIV, according to age recommendations in the summary of the product characteristics (SmPC) and the vaccination protocol established in the Region of Murcia for the 2022–2023 campaign (see Material and Methods section). The comparative analysis between the treatment groups showed statistically significant differences in age, number of siblings, history of infectious diseases and frequency of antibiotic prescription (Table 2). Approximately 17% of children vaccinated with IIV received another concomitant vaccine the same day compared to only 4.4% of children vaccinated with LAIV (*p* < 0.001).

### 3.2. Reasons for Vaccination and Satisfaction with Vaccines

**Figure 1:**
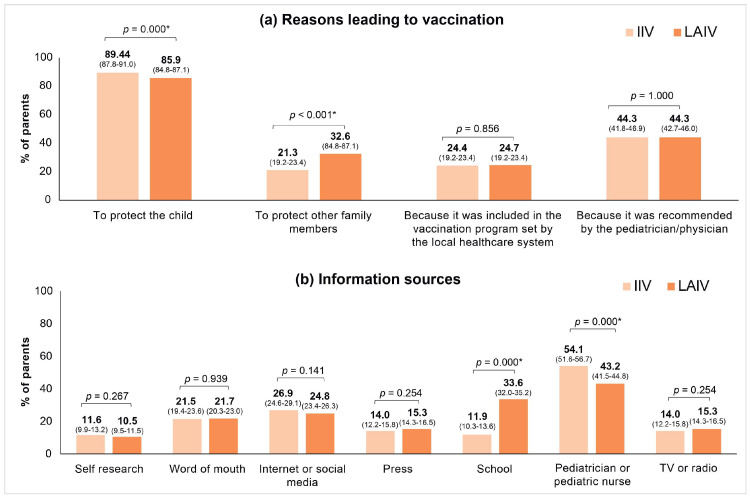
(**a**). Comparative analysis by vaccine of reasons leading parents to vaccinate their children. (**b**) Comparative analysis by vaccine of the information source. IIV, injectable intravenous vaccine; LAIV, live-attenuated intranasal vaccine. * statistically significant values.

VP were asked in the survey the reason for deciding to vaccinate their children, and the most repeated answer in both groups was “To protect the child”, with a significantly higher percentage in IIV (89.4% vs. 85.9; *p* < 0.001). Other reasons for vaccination were “due to recommendation by pediatrician/physician” (44.3% both, *p* = 1.000), “to protect other family members” (21.3% vs. 32.6%, *p* < 0.001) or “due to inclusion in the vaccination program” (24.4% vs. 24.7%, *p* = 0.856) (Figure 1a). The most cited source of information about the influenza vaccination campaign was the pediatrician’s or nurses’ recommendation for both groups, but this was significantly higher in IIV group (54.1% vs. 43.2; *p* < 0.001). Furthermore, we observed that parents of children receiving LAIV were three times more informed at school than parents of children receiving IIV who were informed at kindergarten (33.6% vs. 11.9%, *p* < 0.001) (Figure 1b).

VP were asked about their perception of childhood diseases causing most hospitalizations, choosing pneumonia (51.2%), influenza (33.6%), rotavirus gastroenteritis (8.8%) and meningitis (6.3%).

**Figure 2:**
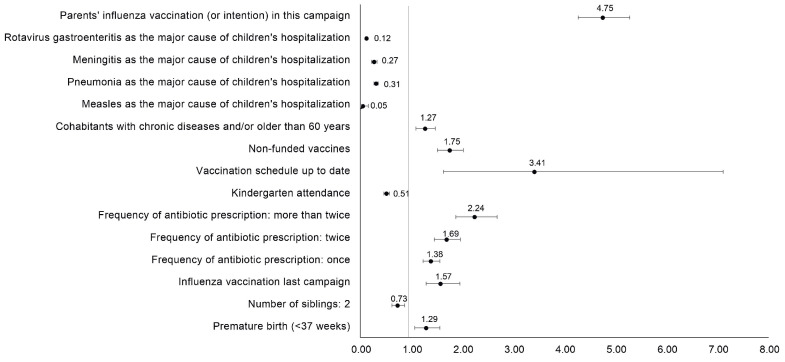
Factors independently associated with vaccination. Odds ratios extracted from the multivariable analysis. The x-axis represents the odds ratio of the y-axis variables with a confidence interval of 95%.

When children’s and parent’s sociodemographic and clinical variables were analyzed by logistic regression, we found that the factors associated with most importance in vaccination decision making were the parent’s vaccination or intention to vaccinate themselves in this campaign (OR = 4.75), followed by compliance with the official vaccination schedule (OR = 3.41) and the prescription of antibiotics more than twice in the previous year (OR = 2.24) (Figure 2). The univariable and multivariable models are depicted in Table 3.

**Figure 3:**
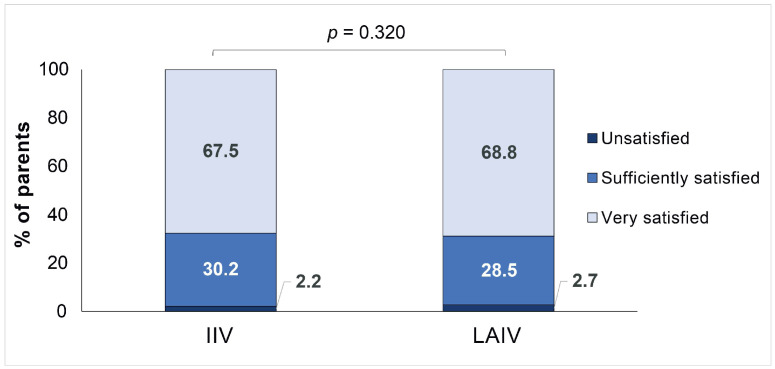
Comparative analysis of parents’ satisfaction between IIV and LAIV. IIV, injectable intravenous vaccine; LAIV, live-attenuated intranasal vaccine. Fisher’s exact test.

**Figure 4:**
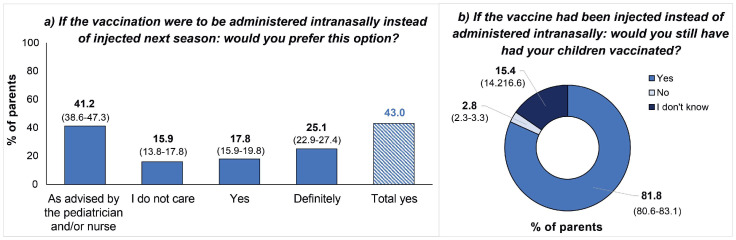
Perspectives of parents for future vaccination campaigns. The column of “total yes” was not part of the original survey; it was calculated post hoc.

Overall, most parents declared that they were very satisfied with the vaccine, and no statistically significant differences between vaccines were observed (IIV: 67.5% vs. LAIV: 68.8%, *p* = 0.320). Only 2.2% and 2.7% of parents, respectively, declared that they were not satisfied with the vaccine (Figure 3). When asked for the route of administration preferred for the next campaign, most parents declared their preference towards LAIV (43.0%), and a remarkable percentage of parents confirmed that they would follow the professional’s advice (41.2%). Moreover, 81.8% of parents stated that they will also have their children vaccinated in the next campaign by injected vaccine vs. 2.8% of them who will not (Figure 4).

### 3.3. Reasons for Non-Vaccination

The NVP of children aged 6 to 23 months declared “lack of healthcare professional recommendation” (30.9%), “lack of information about the vaccination campaign” (21.5%), and “lack of information about the vaccine” (21.0%) as the most important reasons that make them not vaccinate their children. Similarly, the NVP of children aged 24–59 months declared that “they prefer to wait until further experience with the flu vaccine” (22.1%), “their child already contracted the flu” (20.8%) and “lack of recommendation from the healthcare professionals” (17.9%) as the main reasons which lead to no vaccination (Table 4).

The parents of younger children (6–23 months) were less likely to underestimate influenza’s burden in children (8.8%) in contrast to the parents of older children (24–59 months), (15.8%; *p* = 0.000). A remarkable percentage of parents indicated inaccessibility to the vaccination system (8.6% and 10.2%, respectively), and some parents considered the vaccine unsafe (5.4 and 5.5%, respectively). Overall, less than 2% of NVP declared that they do not believe in vaccines.

## 4. Discussion

This study provides insight about the clinical and sociodemographic profile of parents and children eligible for vaccination in the Region of Murcia, suggesting that children’s fragility and parents’ self-experience with vaccination are predominant factors present in vaccinated children, in line with the recent literature.

The uptake of VC aged 6 to 59 months of age was 45.1% of all children registered in the database, exceeding the rates of vaccination in children published up to date in other Spanish regions [9,10,11] and other European countries [12,13,24,25], although these works were undertaken before the new recommendations. It is worth mentioning that even though the desired coverage of 50% was not achieved [20], the results are promising taking into account that it was the first vaccination campaign, and it started in November instead of October. Furthermore, we must consider that the rate of vaccination from one season to another may vary considerably depending on numerous factors [26].

As expected, the children’s clinical context factors such as prematurity, occurrence of acute infections or a high frequency of antibiotic prescriptions were significantly more present among VC, and presented higher odds ratios (OR = 1.29 and OR = 2.24, respectively), suggesting a relationship between the children’s fragility and inclination to vaccinate. Furthermore, our data reveal that the rate of prematurity among VC was slightly higher than the average prematurity rate observed in the Region of Murcia (6.2–7.2%) according to the Spanish National Statistics Institute (INE) [27] and the Regional Statistics Center of Murcia [28]. Chronic disease was not included in these significant variables. Logistic regression confirmed that children who complied with the vaccination schedule or who were prescribed with antibiotics more than twice in the previous year were three times and two times, respectively, more likely to be vaccinated. According to our results, as antibiotics were prescribed more frequently in the previous year, the odds ratios for vaccination in this campaign were higher (Figure 2), supporting the hypothesis that, as a result of previous negative experiences, parents use prophylactic measures to prevent future infections, such as vaccinations. The profile of the VC reported in this study is in line with the homolog study published by Gasparini et al. in 2021 [23]. The parents’ clinical context and past experiences were also determinant for vaccination. We found more parents with chronic disease among the VP group, and they were also more vaccinated in current and past campaigns. Indeed, logistic regression confirmed that parents vaccinated in previous campaigns were slightly more likely to vaccinate their children (OR = 1.57), and those vaccinated in this campaign, or with intention to be vaccinated, were almost five times more likely to vaccinate their children. In line with our data, the previous vaccination of a parent and/or child was already demonstrated to be independently associated with vaccination in a survey study conducted in German parents [17].

It is noteworthy that more VC had cohabitants with chronic disease or older than 60 years, but there were more NVC attending kindergarten. A possible explanation for these results is that parents are more aware of the disease burden in elderly and/or chronically ill people, but not as aware of the disease burden in children. This was already observed in the RELATIVES trial performed in Canada, which reported that 62.1% of parents did not consider that children’s vaccination against influenza was necessary [16]. However, our own data show that the main reason for vaccination was “to protect the child” rather than “to protect other family members”; therefore, we would need a deep sociological understanding to support this hypothesis. Another plausible hypothesis is that higher kindergarten attendance was motivated by busier parents who have potential difficulties in accessing vaccination appointments. The most recent experience of physicians in the studied region confirms that the vaccination coverage is increasing in the kindergarten age after extending school vaccination programs to kindergarten. Even though there are no published data (yet), this experience reveals that easy access to vaccination will increase vaccination coverage, and may explain that fewer vaccinated children attend kindergarten probably because of difficulties in the access to it. Indeed, around 10% of NVP reported inaccessibility to the vaccination system through the survey.

We did not find significant differences in the frequency of vaccination depending on the children’s origin, but there were differences considering the origin of parents (Spanish vs. other countries). These differences were also found in other studies [9,11,29] and may be associated with sociocultural factors and, again, barriers to access to healthcare services.

Almost all surveyed parents in both groups (99.7% and 98.7%) declared that their children were up to date with the vaccination schedule, and 88.3% and 80.1%, respectively, were receptors of non-funded vaccines. This suggests that the decision-making process of influenza vaccination is potentially related to several factors inexistent in the vaccination against other diseases, such as the novelty of the vaccine, lack of information and recommendation, or the limited timeframe of the campaign (approximately three months), which makes accessibility difficult. A recent study conducted in the Netherlands revealed that the parents’ intention of vaccination was strongly related to the perceived importance of the disease, with varicella and influenza worrying them the least [14]. In contrast, our results reveal that most parents rank influenza as the disease causing most hospitalizations in children, second only to pneumonia.

As previously discussed, vaccination decision making was largely motivated by the intention to protect the child in both treatment groups (Figure 1a), but it is remarkable that the parents of children vaccinated with LAIV were significantly more conscious of protecting other family members. These differences may be derived from the children’s age, since LAIV-vaccinated children were older, and parents’ awareness may be slightly lower and more focused on other family members with a potentially higher disease burden. Furthermore, as children grow older, they have fewer vaccination visits to the medical center as they complete their vaccination schedules, and therefore have fewer opportunities to receive the influenza vaccine over time. This may be an extra effort for parents who are less aware of the need for vaccination for the reasons mentioned above. This profile observed in the VP of older children would justify competent health institutions’ facilitation of vaccination with LAIV, as well as school vaccination.

Overall, parents received information about the campaign mostly from healthcare professionals (pediatrician or pediatric nurses), but additionally, parents of children vaccinated with LAIV placed “school” as the second source of information, approximately three times higher in comparison to IIV. These differences must be considered cautiously since many children vaccinated with IIV were still too young for schooling, even in kindergarten. These considerations are included in this study’s limitations.

The causes of non-vaccination most repeated in the survey specific for NVP were a lack of recommendation from healthcare professionals (first cause for the parents of younger children and third cause for the parents of older children), lack of information about the campaign and/or vaccine, or reticence to vaccinate the child until further experience with the vaccine. Additionally, it should be noted that the percentage of parents stating that they do not believe in vaccines was only 2%. This percentage was calculated considering only NVP, and it might be reduced approximately to half if considering all surveyed parents (VP and NVP), leading us to conclude that the population under study considers influenza vaccines reliable.

The primary source of information for VP was the physician or nurse, suggesting that the recommendation of healthcare professionals is crucial in the parents’ decision-making process, as has been corroborated in many studies [17,30,31]. The lack of recommendation suggests that a significant percentage of health professionals are not fully aware of the burden of disease in children. As mentioned, around 10% of surveyed NVP stated the impossibility to obtain an appointment for vaccination. This fact highlights the importance of improving vaccination access through school vaccination, which has been shown to be feasible and effective [32]. Moreover, two pilot studies in Galicia confirmed a 5% increase in children’s vaccination by promoting mass vaccination in designated sites with long shifts of vaccination by imitating the SARS-COV-2 vaccination campaigns [33]. Our data revealed that a remarkably high percentage of NVP (20.8%) had an erroneous idea about vaccination, declaring that they have not vaccinated their children because they had already contracted influenza that year, which highlights the fact that parents and patients overall are insufficiently informed. It is of the utmost importance to address this misinformation in order to achieve better coverage in future campaigns.

When evaluating the opinion of parents, our results showed that most were very satisfied with both vaccinees (IIV, 67.5% and LAIV, 68.8%), but the analysis could not conclude a significant difference between them. Nevertheless, we observed that parents preferred the LAIV option for the next vaccination campaign, agreeing with other studies which reported similar percentages of preference towards LAIV: 83.0% in the cross-sectional cohort of parents surveyed in Bologna (Italy) [13]; 83.8% in an observational study conducted in Milan (Italy) [23]; and 75% in the RELATIVES trial conducted in Canada [16].

The survey methodology entails advantages and limitations. Firstly, the survey was designed to obtain relevant information that could not be obtained by reviewing the electronic health records, such as a lot of sociodemographic data from parents and children or the parents’ opinion. Furthermore, by using a survey, the investigators ensured that there was no missing information in the electronic records, which is common in retrospective observational studies. Despite these advantages, the survey design is not exempt of limitations, introducing systematic errors in the data collection derived from the self-reporting method and recall bias. The questions in the survey were specifically designed in friendly and easy language to avoid misunderstanding and further errors in data collection. Secondly, we must take into account that there is a major limitation in the data collection design, as it does not allow us to know if the same parent has included his/her data more than once when reporting for more than one child. As required by the local and European authorities, all data must be anonymized, and we could not identify these registries thereafter. Considering the data analysis, there is an age bias in the comparative analysis between the vaccines studied here. Following the indications on the product SmpC and the regional vaccination protocol, the IIV was only administered to younger children (6 to 23 months old), whilst LAIV was administered to older children (24 to 29 months old). We are aware that this bias may influence some of the analysis performed, such as the parents’ preference, but this strictly reflects the current routine practice followed by physicians in the Region of Murcia, Spain. In line with the age bias, the statistically significant differences found in some variables studied, for example, in the influenza vaccination in the last campaign, must be considered with caution because children receiving IIV could have been out of the indicated age in the last campaign. One of the limitations found in the survey per se is that parents were not asked about the number of times they attended the primary care center during the vaccination campaign, because an elevated number of visits in prematurely born children, children with chronic disease or children indicated to receive LAIV (24–59 months of age) may have had an impact on the child’s chances of being vaccinated.

To our knowledge, this is, to date, the only comparative analysis performed in Spain in a large cohort of participants after the universal vaccination recommendation, which provides insight about the pediatric vaccination status and the profile of both VP and NVP. Additionally, we provide novel data on the comparison of vaccine preference depending on the route of administration.

## 5. Conclusions

The vaccination coverage achieved in the 2022–2023 season was acceptable and promising. The clinical context of parents and children was determinant in vaccination decision making, especially previous and current vaccination of parents. Most parents indicated high satisfaction with vaccination, with no significant differences among vaccines, but with a remarkable inclination for LAIV administration for future campaigns.

The parents considered the healthcare professional’s opinion and recommendation as the most valuable source of information; thus, it is of the utmost importance to increase the involvement of primary care doctors, nurses, and pediatricians in informing parents.

## Figures and Tables

**Table 1:** Descriptive and comparative analysis between vaccinated and non-vaccinated children and parents.

Children’s Characteristics	Vaccinated Children (VC) N = 4971	Non-Vaccinated Children (NVC) N = 5028	*p* Value
% of children	49.7%	50.3%	-
Age (years), median (IQR)			<0.001
6–12 months, n (%)	499 (10.4)	565 (11.24)	
1 year, n (%)	938 (18.9)	954 (19.0)	
2 years, n (%)	1176 (23.7)	1001 (19.9)	
3 years, n (%)	1173 (23.6)	1097 (21.8)	
4 years, n (%)	1182 (23.8)	1411 (28.1)	
Sex (male), n (%)	2568 (51.8)	2607 (51.9)	0.952
Premature birth (<37 weeks), n (%)	427 (8.7)	322 (6.4)	<0.001
Country of origin, n (%)			0.277
Spain	4918 (98.9)	4690 (98.6)	
Other	55 (1.1)	68 (1.4)	
Siblings, n (%)			<0.001
None	1956 (39.5)	1926 (38.3)	
1	2302 (46.4)	2217 (44.1)	
2	555 (11.2)	682 (13.6)	
>2	144 (2.9)	203 (4.0)	
History of acute infectious disease requiring medical attention in the previous year (2022), n (%)	2691 (54.3)	2337 (46.5)	<0.001
Frequency of antibiotic prescription in the past year, n (%)			<0.001
None	1920 (38.7)	2385 (47.4)	
1	1539 (31.0)	1551 (30.9)	
2	852 (17.2)	701 (13.9)	
>2	654 (13.2)	391 (7.8)	
Kindergarten attendance to, n (%)	2256 (58.9)	3536 (70.3)	<0.001
Age (months) at kindergarten initiation, median (IQR)	13.3 (13.1–13.6)	14.3 (14.0–14.6)	0.004
Completed vaccination schedule according to age, n (%)	4945 (99.7)	4962 (98.7)	<0.001
Non-funded vaccines, n (%)	4376 (88.3)	4027 (80.1)	<0.001
Influenza vaccination in the last campaign (2021–2022), n (%)	612 (12.6)	232 (4.6)	<0.001
Chronic ^(1)^ disease, n (%)	219 (4.4)	189 (3.8)	0.106
**Parents’ characteristics**	**Vaccinated** **N = 4971**	**Non-Vaccinated** **N = 5028**	***p* Value**
Age range, n (%)			
<20	30 (0.6)	21 (0.4)	0.206
20–29	360 (7.2)	538 (10.5)	<0.001
30–39	3059 (61.5)	2833 (55.3)	<0.001
40–49	1502 (31.2)	1599 (31.2)	0.290
≥50	30 (0.6)	56 (1.1)	0.009
Country of origin, n (%)			
Spain	4483 (91.0)	4350 (86.5)	<0.001
Other	428 (9.0)	678 (13.5)	
Education, n (%)			
None	26 (0.5)	29 (0.6)	<0.001
Primary education	332 (6.7)	258 (5.1)	
Secondary education	1753 (35.4)	1933 (38.4)	
Higher education	2848 (57.4)	2808 (55.9)	
Chronic ^(1)^ disease, n (%)	915 (18.5)	777 (15.5)	<0.001
Parents’ influenza vaccination in the last campaign (2021–2022), n (%)	2105 (42.6)	1237 (24.6)	<0.001
Parents’ influenza vaccination (or intention) in the current campaign (2022–2023), n (%)	2628 (53.2)	925 (18.4)	<0.001
Cohabitants with chronic disease ^(1)^ and/or older than 60 years, n (%)	739 (15.0)	480 (9.6)	<0.001

^(1)^ Chronic disease: cancer, immune system deficiency, genetic disease, allergic disease, autoimmune disease, cardiovascular disease, endocrine disease, gastrointestinal disease, neurologic disease, respiratory disease, renal disease.

**Table 2:** Descriptive and comparative analysis among vaccinated children by vaccine.

Children’s Characteristics	IIV N = 1437		LAIV N = 3531		*p* Value
Children	n (%)	(95% CI)	n (%)	(95% CI)	
Age (years), median (IQR)					<0.001
6–12 months, n (%)	499 (34.7)	(32.3–37.2)	-	-	
1 year, n (%)	938 (65.3)	(62.8–67.7)	-	-	
2 years, n (%)	-	-	1176 (33.3)	(31.8–34.9)	
3 years, n (%)	-	-	1173 (33.2)	(31.7–34.8)	
4 years, n (%)	-	-	1182 (33.5)	(31.9–35.0)	
Sex (male), n (%)	738 (51.5)	(49.0–54.1)	1830 (51.9)	(50.2–53.5)	0.851
Premature birth (<37 weeks), n (%)	114 (8.0)	(6.6–9.4)	313 (8.9)	(8.0–9.0)	0.289
Country of origin ^(1)^, n (%)					0.161
African Region	1 (0.1)	(0.0–0.2)	1 (0.0)	(0.0–0.1)	
Region of the Americas	4 (0.3)	(0.0–0.6)	22 (0.6)	(0.4–0.9)	
South East Asian Region	0 (0.0)	-	2 (0.1)	(0.0–0.1)	
European Region ^(2)^	0 (0.0)	-	8 (0.2)	(0.1–0.4)	
Spain	1425 (99.7)	(99.3–100.0)	3493 (99.0)	(98.7–99.3)	
Eastern Mediterranean Region	0 (0.0)	-	0 (0.0)	-	
Western Pacific Region	0 (0.0)	-	2 (0.1)	(0.0–0.1)	
Siblings, n (%)					<0.001
None	730 (50.9)	(48.4–53.5)	1226 (34.8)	(33.2–36.4)	
1	534 (37.3)	(34.8–39.8)	1768 (50.2)	(48.5–51.8)	
2	134 (9.4)	(7.8–10.9)	421 (12.0)	(10.9–13.0)	
>2	35 (2.4)	(1.6–3.2)	109 (3.1)	(2.5–3.7)	
History of acute infectious disease requiring medical attention in the previous year (2022), n (%)	707 (49.4)	(46.8–52.0)	1984 (56.3)	(54.7–57.9)	<0.001
Frequency of antibiotic prescription in the past year, n (%)					<0.001
None	774 (53.8)	(51.3–56.4)	1146 (32.5)	(31.0–34.0)	
1	396 (27.5)	(25.2–29.9)	1143 (32.4)	(30.9–34.0)	
2	157 (10.9)	(9.3–12.5)	695 (19.7)	(18.4–21.0)	
>2	111 (7.7)	(6.3–9.1)	543 (15.4)	(14.2–16.6)	
Kindergarten attendance to, n (%)	742 (51.7)	(49.1–54.3)	1514 (63.3)	(61.4–65.3)	<0.001
Age (months) at kindergarten initiation, median (IQR)	10.0 (7.0)		13.0 (11.0)		<0.001
Completed vaccination schedule according to age, n (%)	1429 (99.6)	(99.3–99.9)	3516 (99.8)	(99.7–100.0)	0.218
Non-funded vaccines, n (%)	1317 (91.8)	(90.4–93.3)	3059 (86.9)	(85.8–88.0)	<0.001
Influenza vaccination in the last campaign (2021–2022), n (%)	58 (4.1)	(3.1–5.1)	563 (16.0)	(14.8–17.2)	<0.001
Chronic disease ^(3)^, n (%)	42 (2.9)	(2.1–3.8)	177 (5.0)	(4.3–5.8)	0.001
**Parents**					
Age range, n (%)					
<20	11 (0.3)	(0.1–0.5)	19 (1.3)	(0.7–1.9)	<0.001
20–29	201 (5.7)	(4.9–6.5)	159 (11.0)	(9.4–12.7)	
30–39	2071 (58.6)	(57.0–60.3)	988 (68.6)	(66.2–71.0)	
40–49	1224 (34.7)	(33.1–36.2)	278 (19.3)	(17.3–21.3)	
≥50	28 (0.8)	(0.5–1.1)	2 (0.1)	(0.0–0.3)	
Sex (female), n (%)	1304 (90.9)	(89.4–92.4)	3272 (92.9)	(92.1–93.8)	0.016
Country of origin ^(1)^, n (%)					
African Region	2 (0.1)	(0.0–0.3)	7 (0.2)	0.1–0.4)	0.915
Region of the Americas	91 (6.4)	(5.1–7.6)	231 (6.6)	(5.8–7.4)	
South East Asian Region	0 (0.0)	-	2 (0.1)	(0.0–0.1)	
European Region ^(2)^	29 (2.0)	(1.3–2.8)	55 (1.6)	(1.2–2.0)	
Spain	1302 (91.0)	(89.5–92.5)	3183 (91.0)	(90.0–91.9)	
Eastern Mediterranean Region	7 (0.5)	(0.1–0.9)	19 (0.5)	(0.3–0.8)	
Western Pacific Region	0 (0.0)	-	2 (0.1)	(0.0–0.1)	
Education, n (%)					
None	7 (0.5)	(0.1–0.9)	19 (0.5)	(0.3–0.8)	0.006
Primary education	73 (5.1)	(4.0–6.2)	259 (7.4)	(6.5–8.2)	
Secondary education	488 (34.0)	(31.5–36.4)	1265 (35.9)	(34.3–37.5)	
Higher education	868 (60.5)	(57.9–63.0)	1980 (56.2)	(54.6–57.8)	
Chronic disease ^(3)^, n (%)	259 (18.0)	(16.0–20.0)	656 (18.6)	(17.3–19.9)	0.657
Parents’ influenza vaccination in the last campaign (2021–2022), n (%)	741 (51.7)	(49.1–54.3)	1364 (38.9)	(37.3–40.5)	<0.001
Parents’ influenza vaccination (or intention) in the current campaign (2022–2023), n (%)	776 (54.1)	(51.5–56.7)	1852 (52.8)	(51.1–54.5)	0.414
Cohabitants with chronic disease ^(3)^ and/or older than 60 years, n (%)	196 (13.7)	(11.9–15.5)	543 (15.5)	(14.3–16.7)	0.113

^(1)^ According to WHO division. ^(2)^ European Region without Spain. ^(3)^ Chronic disease: cancer, immune system deficiency, genetic disease, allergic disease, autoimmune disease, cardiovascular disease, endocrine disease, gastrointestinal disease, neurologic disease, respiratory disease, renal disease.

**Table 3:** Univariable and multivariable analysis extracted from the logistic regression model used to estimate the factors independently associated with vaccination.

Variables	Univariable Analysis		Multivariable Analysis	
	OR (95% CI)	*p* Value	OR (95% CI)	*p* Value
Premature birth (<37 weeks) ^(1)^	1.38 (1.19–1.61)	<0.001	1.29 (1.07–1.56)	0.0078
Number of siblings ^(2)^, n (%)				
1	1.02 (0.94–1.11)	0.613	0.93 (0.84–1.04)	0.198
2	0.80 (0.7–0.91)	<0.001	0.73 (0.62–0.87)	0.0002
>2	0.70 (0.56–0.87)	<0.001	0.77 (0.58–1.02)	0.0679
Influenza vaccination last campaign (child) ^(3)^	2.97 (2.54–3.47)	<0.001	1.57 (1.29–1.95)	<0.0001
Acute infectious disease in 2022 ^(4)^	1.37 (1.26–1.48)	<0.001	1.31 (1.02–1.26)	0.0207
Frequency of antibiotic prescription ^(2)^, n (%)				
Once	1.23 (1.12–1.35)	<0.001	1.38 (1.23–1.56)	<0.0001
Twice	1.51 (1.34–1.70)	<0.001	1.69 (1.45–1.96)	<0.0001
More than twice	2.08 (1.81–2.39)	<0.001	2.24 (1.87–2.68)	<0.0001
Kindergarten attendance ^(5)^	0.61 (0.55–0.66)	<0.001	0.51 (0.46–0.57)	<0.0001
Up-to-date vaccination schedule ^(6)^	5.06 (2.79–9.18)	<0.001	3.41 (1.63–7.11)	0.0011
Non-funded vaccines ^(7)^	1.88 (1.68–2.10)	<0.001	1.75 (1.51–2.02)	<0.0001
Cohabitants with chronic disease ^(a)^ and/or older than 60 years ^(8)^	1.67 (1.48–1.88)	<0.001	1.27 (1.09–1.47)	0.0024
Personal opinion about childhood diseases causing most hospitalizations ^(9)^		<0.001		
Measles	0.06 (0.02–0.18)	<0.001	0.05 (0.02–0.16)	<0.0001
Pneumonia	0.30 (0.26–0.33)	<0.001	0.31 (0.27–0.35)	<0.0001
Meningitis	0.23 (0.19–0.28)	<0.001	0.27 (0.22–0.33)	<0.0001
Rotavirus gastroenteritis	0.14 (0.12–0.16)	<0.001	0.12 (0.10–0.14)	<0.0001
Influenza vaccination in the previous year (parent) ^(10)^	2.27 (2.09–2.48)	<0.001	-	-
Influenza vaccination (or intention) this campaign (parent) ^(11)^	5.04 (460–5.52)	<0.001	4.75 (4.27–5.28)	<0.0001
Chronic ^(a)^ disease (parent) ^(12)^	1.24 (1.11–1.37)	<0.001	-	-

Reference categories: ^(1)^ no premature birth (≥37 weeks); ^(2)^ none; ^(3)^ no influenza vaccination last campaign; ^(4)^ no acute infectious disease in 2022; ^(5)^ no kindergarten attendance; ^(6)^ no up-to-date vaccination schedule; ^(7)^ no non-funded vaccines; ^(8)^ no cohabitants with chronic disease and/or older than 60 years; ^(9)^ influenza vs. measles, pneumonia, meningitis and rotavirus gastroenteritis; ^(10)^ no influenza vaccination in the previous year (parent); ^(11)^ no influenza vaccination (or intention) this campaign (parent); ^(12)^ no chronic disease (parent). The variables “Influenza vaccination last year (parents)” and “Chronic disease (parent)” were no longer significant when adjusted with the rest of the variables in the multivariable model. Variables with statistical significance *p* < 0.2 in the univariable logistic regression analysis were considered significant and included in a multivariable model. Test results with *p* < 0.05 in the multivariable analysis are considered statistically significant. ^(a)^ Chronic disease: cancer, immune system deficiency, genetic disease, allergic disease, autoimmune disease, cardiovascular disease, endocrine disease, gastrointestinal disease, neurologic disease, respiratory disease, renal disease.

**Table 4:** Comparative analysis of reasons leading to no vaccination stratified by children’s age.

Reasons for No Vaccination	Age 6–23 Months N = 1519		Age 24–59 Months N = 3509		*p* Value
	n (%)	95% CI	n (%)	95% CI	
The healthcare professional of reference did not recommend it	469 (30.9)	28.6–33.2	629 (17.9)	16.7–19.2	<0.001
I would rather wait until further experience with the flu vaccine	270 (17.8)	15.9–19.7	774 (22.1)	20.7–23.4	<0.001
My son/daughter has contracted flu this year	214 (14.1)	12.3–15.8	728 (20.8)	19.4–22.1	<0.001
Lack of information about the vaccination campaign	327 (21.5)	19.5–23.6	528 (15.1)	13.9–16.2	<0.001
Lack of information about the vaccine	319 (21.0)	19.0–23.1	575 (16.4)	15.2–17.6	<0.001
I consider flu an infection of minor importance in children <5 years	133 (8.8)	7.3–10.2	553 (15.8)	14.6–17.0	<0.001
Inaccessibility (i.e., impossibility to get an appointment)	130 (8.6)	7.2–10.0	358 (10.2)	9.2–11.2	0.078
The vaccine did not demonstrate effectiveness	107 (7.0)	5.8–8.3	339 (9.7)	8.7–10.6	0.002
I consider the flu vaccine unsafe	82 (5.4)	(4.3–6.5)	193 (5.5)	(4.8–6.3)	0.946
Influence of friends’/family’s opinion	66 (4.3)	(3.3–5.4)	163 (4.7)	(4.0–5.3)	0.659
I do not believe in vaccines	16 (1.1)	0.5–1.6	65 (1.9)	1.4–2.3	0.038

## Data Availability

The data that support the findings of this study are available on request from the corresponding author.

## Supplementary Materials

The following supporting information can be downloaded at: https://www.mdpi.com/article/10.3390/vaccines12020192/s1, Figure S1: Data collection chart.
