# Supplementary figures and images for: Influenza Vaccination in Children Younger than 5 Years in the Region of Murcia (Spain), a Comparative Analysis among Vaccinating and Non-Vaccinating Parents: Data from the FLUTETRA Study
Source: Vaccines (Basel). 2024 Feb 13;12(2):192. doi: 10.3390/vaccines12020192 (PMC10892024; doi:10.3390/vaccines12020192)

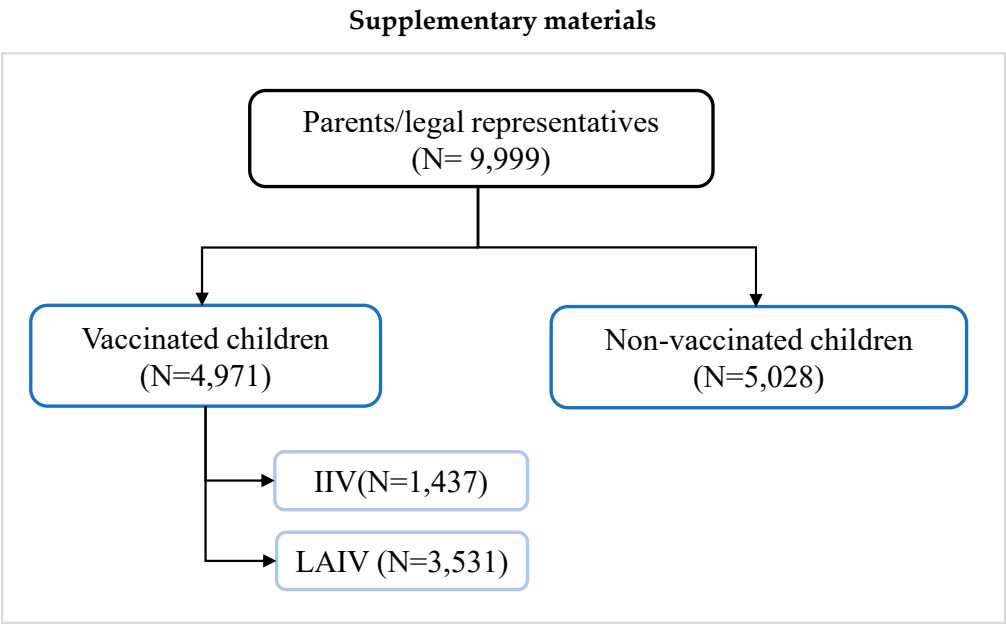

**Figure S1.** Data collection chart

Supplement: Supplementary file 1 [file vaccines-12-00192-s001.zip › vaccines-2853418-supplementary.pdf]
